# LGBTQIA+ People’s Perspectives on LGBTQIA+-Targeted State Policies and Mental Health: A Qualitative Study

**DOI:** 10.1001/jamanetworkopen.2025.46538

**Published:** 2026-01-02

**Authors:** Briana S. Last, Madeline Poupard, Noah Williamson, Laura Jans, Akshita Arora, Nguyen K. Tran, Juno Obedin-Maliver, Mitchell R. Lunn, Annesa Flentje

**Affiliations:** 1 Department of Psychology, Stony Brook University, Stony Brook, New York; 2 The PRIDE Study/PRIDEnet, Stanford University School of Medicine, Palo Alto, California; 3 Department of Obstetrics & Gynecology, Stanford University School of Medicine, Stanford, California; 4 Department of Epidemiology and Population Health, Stanford University School of Medicine, Stanford, California; 5 Division of Nephrology, Department of Medicine, Stanford University School of Medicine, Stanford, California; 6 Stanford Prevention Research Center, Department of Medicine, Stanford University School of Medicine, Stanford, California; 7 Alliance Health Project, Department of Psychiatry and Behavioral Sciences, School of Medicine, University of California, San Francisco

## Abstract

**Importance:**

There has been a rise in state policies targeting the rights of lesbian, gay, bisexual, transgender, queer, intersex, asexual, and other sexual and gender minority (LGBTQIA+) people in the United States. Although large-scale studies have quantified associations between these policies and LGBTQIA+ people’s mental health, less research has centered the first-hand accounts of LGBTQIA+ people.

**Objective:**

To examine LGBTQIA+ people’s accounts of how they perceive these policies to be impacting their mental health.

**Design, Setting, and Participants:**

From July to October 2024, 1-hour, semistructured, virtual interviews were conducted with LGBTQIA+ adults living in states that had recently proposed or enacted state LGBTQIA+-targeted policies. The state policies included: (1) gender-affirming care restrictions; (2) sports bans for transgender and nonbinary (TNB) people; (3) public bathroom bans for TNB people; (4) school restrictions of sexual orientation and/or gender identity discussions; and (5) religious exemptions, which permit individuals and service organizations to withhold services from LGBTQIA+ people for religious reasons.

**Main Outcomes and Measures:**

Transcripts were analyzed using thematic analysis, focusing on participants’ perceptions of the mental health impacts of these policies.

**Results:**

Interviews with the 61 adult participants in the sample (median [IQR] age, 35 [30-48] years; 13 cisgender men [21.3%], 19 cisgender women [31.1%], 16 nonbinary people [26.2%], 8 transgender men [13.1%], 2 transgender women [3.3%], and 3 people with another gender identity [4.9%]) revealed that LGBTQIA+ people perceive these policies to negatively impact their mental health. These perceived impacts were organized into 3 themes: (1) chronic worry and hypervigilance, (2) social isolation, and (3) hopelessness and powerlessness. A fourth cross-cutting theme was also identified: participants perceived these policies’ mental health impacts to be unequal and more pronounced for those most frequently targeted by these policies (eg, youths, TNB people), racially and ethnically minoritized people, those without social and financial resources, and those living in rural areas.

**Conclusions and Relevance:**

In this qualitative study, LGBTQIA+ adults in the United States perceived LGBTQIA+-targeted policies to have profound and unequal impacts on their mental health. As LGBTQIA+-targeted policies increase in number, multilevel resources and supports are necessary to support LGBTQIA+ people’s well-being.

## Key Points

**Question:**

What are lesbian, gay, bisexual, transgender, queer, intersex, asexual, and other sexual and gender minority (LGBTQIA+) people’s perceptions of the mental health impacts of recent US LGBTQIA+-targeted policies?

**Findings:**

In this qualitative study, 61 LGBTQIA+ adults living in states that recently proposed or enacted LGBTQIA+-targeted policies were interviewed. Participants described 3 perceived mental health impacts, including chronic worry and hypervigilance, social isolation, and hopelessness and powerlessness; they also described that these policies’ perceived negative mental health impacts are more pronounced for those most targeted (transgender and nonbinary people, youths) and for socially and economically marginalized and geographically isolated communities.

**Meaning:**

In this study, LGBTQIA+ people perceived LGBTQIA+-targeted policies to negatively impact their mental health.

## Teaser

This qualitative study examines how lesbian, gay, bisexual, transgender, queer, intersex, asexual, and other sexual and gender minority (LGBTQIA+) adults perceive how their mental health has been affected by policies targeting LGBTQIA+ individuals, including, among others, restrictions on gender-affirming care, participation in sports for transgender and nonbinary individuals, discussion of sexual orientation or gender identity in schools.

## Introduction

US state and federal policies targeting lesbian, gay, bisexual, transgender, queer, intersex, asexual, and other sexual and gender minority (LGBTQIA+) people are on the rise.^1^ Starting in 2021, each year has surpassed the last year’s record for the greatest number of LGBTQIA+-targeted bills proposed across state legislatures.^1,2^ Since taking office, the Trump administration has rescinded several antidiscrimination protections for LGBTQIA+ people and issued executive orders restricting transgender and nonbinary (TNB) people’s rights and health care access.^3^ The Supreme Court has expanded the free speech, religious, and parental rights of those opposed to LGBTQIA+ equity.^4,5^ These state and federal policies affect many aspects of LGBTQIA+ people’s lives, including their access to public accommodations, health care, social services, and public education on sexual orientation and gender identity.^2^

These policies are associated with worse mental health among LGBTQIA+ people^6,7,8,9,10^ and are mediated through complex psychosocial pathways.^11^ Theoretical models and frameworks, such as the social determinants of health framework (which traces how social factors determine well-being),^12,13^ the minority stress model (which describes how stressors unique to minoritized groups can harm their mental health),^11^ and the social safety perspective (which posits that discriminatory experiences deprive people of the human need for social connection and belonging),^14^ elucidate some of these potential pathways. LGBTQIA+-targeted policies may worsen mental health by depriving LGBTQIA+ people of basic needs and resources (health care, social services),^15^ instilling fear that LGBTQIA+ people will lose additional rights,^9,16,17^ and licensing discrimination and harassment through the policies’ expressive function—that is, signaling societal values, establishing acceptable behaviors, and shaping public attitudes about who should be afforded rights and respect.^16,18,19,20,21,22^ For example, one study of more than 72 000 college students found that the mere proposal of LGBTQIA+-targeted policies was associated with increased depressive symptoms.^20^ Similarly, among TNB adults living in a state that had not enacted LGBTQIA+-targeted policies, those who feared losing their rights experienced heightened depression and anxiety.^16^

Given the recent increase in LGBTQIA+-targeted policies, examining how LGBTQIA+ people are psychologically experiencing this new policy environment is critical. Although quantitative studies help establish measurable relationships between policies and mental health, they must be considered alongside LGBTQIA+ people’s diverse lived experiences. Qualitative studies elucidate how people perceive policies to influence their lives in multifaceted and dynamic ways not easily captured by standardized quantitative measures. Qualitative studies can also characterize the complex psychosocial pathways through which policies may relate to mental health and the intersecting social forces (eg, economic inequality, racial oppression) that uniquely shape and texture people’s experiences of these policies.^23,24,25^ Incorporating how LGBTQIA+ people describe and make meaning of their experiences can enhance advocacy and intervention efforts designed to improve their resilience and well-being.^26^ In the present study, we leveraged qualitative methods to examine the perspectives of LGBTQIA+ individuals living in states that had recently proposed or enacted LGBTQIA+-targeted policies. We aimed to understand: (1) LGBTQIA+ people’s perceptions of the direct and indirect mental health effects of these policies and (2) how these perceived effects are textured by the social forces and contextual factors that shape LGBTQIA+ people’s lives.

## Methods

The Stony Brook University Institutional Review Board approved the study. The study materials are publicly available.^27^ The Consolidated Criteria for Reporting Qualitative Research (COREQ) reporting guideline guided our study reporting.^28^

### LGBTQIA+-Targeted Policies

We selected LGBTQIA+-targeted policies using the Movement Advancement Project’s database^29^ including (1) gender-affirming care (GAC) restrictions; (2) sports bans for TNB people; (3) bathroom bans for TNB people; (4) restrictions on school discussions of sexual orientation and/or gender identity; and (5) religious exemption policies, which permit individuals and social service organizations to withhold services from LGBTQIA+ people for religious reasons. We chose to focus on state policies that (1) were recently enacted or proposed (eg, GAC restrictions), so that participants could describe their perspectives of these policies’ introduction and/or passage, and (2) were enacted in at least 10 states to capture variation in participants’ experiences of policy implementation across states.

### Procedures

Participants were purposively sampled from The PRIDE Study, a community-engaged, dynamic, prospective cohort study of LGBTQIA+ US adults’ health.^30,31,32^ To be eligible for this study, participants must have completed the 2023 PRIDE Study annual questionnaire. We recruited participants living in states that (1) proposed but did not enact any LGBTQIA+-targeted policies described previously; (2) enacted some but not all these policies; or (3) enacted all these policies. We randomly sampled participants in each category, oversampling racially and ethnically minoritized and TNB people.

We recruited participants in waves, adjusting how many participants we invited from each sampling category depending on what we learned in interviews.^33^ We initially invited equal numbers of participants (n = 30) from each sampling category, but quickly learned that participants in states that only proposed (not enacted) LGBTQIA+-targeted policies seldom provided in-depth reports on how the proposed policies shaped their lives. Given the focus of qualitative research on depth over breadth, after this first wave, we exclusively sampled participants in states that enacted some or all policies under study. We recruited until we achieved information power.^33^ That is, we interviewed participants until we collected sufficient data from diverse participants from across the country who provided high-quality, in-depth responses to our research questions.^33^

Interested participants completed an online consent form and 10-minute online survey, which collected participants’ sociodemographic information (eg, age, gender identity, sexual orientation, identification with nonmutually exclusive race and ethnicity categories), and interview availability. Individuals who completed the 10-minute online survey preceding the interview provided electronic informed consent. At the beginning of each interview, participants also provided verbal informed consent. Between July and October 2024, our team (faculty, graduate students, and research assistants) verbally consented participants and conducted individual, virtual interviews, which lasted a mean (SD) of 57.9 (12.1) minutes, using a semistructured guide. Before conducting interviews, we sought feedback on the guide from The PRIDE Study participant and research advisory committees; we also piloted it with 3 LGBTQIA+ adults. Throughout interviews, we kept field notes and met to discuss our impressions and adjust our sampling strategy. We audio recorded and transcribed interviews; at least 3 trained team members (including the interviewer) independently validated the transcriptions. Participants received a $50 gift card.

### Data Analysis

We descriptively characterized participants’ sociodemographic characteristics. We analyzed interview transcripts using thematic analysis in MAXQDA Plus 24 (VERBI).^34,35^ First, we independently reviewed the transcripts, took field notes, and developed preliminary codes that reflected participants’ shared experiences. Second, we met as a group to share our notes and preliminary codes. Third, we analyzed 7 transcripts together, line-by-line, generating codes inductively and deductively. Throughout line-by-line coding, we grouped the codes into preliminary themes by identifying shared patterns of meaning across codes. Fourth, we organized the codes and themes further into a codebook. Fifth, we divided the remaining 54 transcripts among our team of 6 coders. We independently coded transcripts using the codebook. During this stage, we met regularly to ensure consistent codebook application. Sixth, once independent coding was complete, the first and second author reviewed and validated the entire team’s coding to ensure consistent codebook application, meeting weekly to resolve discrepancies via consensus discussion. Seventh, our entire coding team finalized our conceptualization of the themes, their interrelationships, and their names. Given the myriad topics discussed in the interviews (eg, participants’ attitudes about the political landscape, perceptions of the motivations behind these policies, and political engagement) and the number of themes that we identified during the coding process, our team decided to focus the present analysis on a subset of health-related themes that cohered together (policies’ perceived mental health impacts).

## Results

Sixty-one adults completed interviews (median [IQR] age, 35 [30-48] years; 13 cisgender men [21.3%], 19 cisgender women [31.1%], 16 non-binary people [26.2%], 8 transgender men [13.1%], 2 transgender women [3.3%], and 3 people with another gender identity [4.9%]). Participants were racially and ethnically diverse: 8 identified as American Indian or Alaska Native (13.1%); 7 as Asian (11.5%); 10 as Black, African American, or African (16.4%); 7 as Hispanic, Latinx, or Spanish (11.5%); 1 as Native Hawaiian or Other Pacific Islander (1.6%); 47 as White (77.0%); and 3 who indicated “None of these fully describe me” (4.9%) (Table 1). Thirty participants (49.2%) lived in states that had enacted all policies under study, 27 (44.2%) lived in states that had enacted some policies, and 4 (6.6%) lived in states that had proposed but not enacted these policies. Participants lived in the South (37 [60.7%]), Northeast (7 [11.5%]), Midwest (8 [13.1%]), and West (9 [14.8%]) (Figure).

**Table 1.** Participant Characteristics

Characteristic	Participants, No. (%) (N = 61)
State sampling	
Enacted all policies	30 (49.2)
Enacted some policies	27 (44.3)
Proposed 1 or more policies	4 (6.6)
State Census region	
South	37 (60.7)
Northeast	7 (11.5)
Midwest	8 (13.1)
West	9 (14.8)
Age, median (IQR), y	35 (30-48)
Racial and ethnic identity^a^	
American Indian or Alaska Native	8 (13.1)
Asian	7 (11.5)
Black, African American, or African	10 (16.4)
Hispanic, Latinx, or Spanish	7 (11.5)
Native Hawaiian or Other Pacific Islander	1 (1.6)
White	47 (77.0)
None of these fully describe me^b^	3 (4.9)
Sexual orientation	
Asexual, demisexual, or gray-ace	4 (6.6)
Bisexual or pansexual	19 (31.1)
Gay or lesbian	21 (34.4)
Queer	16 (26.2)
Straight or heterosexual	1 (1.6)
Gender identity	
Cisgender man	13 (21.3)
Cisgender woman	19 (31.1)
Nonbinary	16 (26.2)
Transgender man	8 (13.1)
Transgender woman	2 (3.3)
Another gender identity	3 (4.9)
Household income, $	
1-10 000	3 (4.9)
10 001-20 000	8 (13.1)
20 001-30 000	4 (6.6)
30 001-40 000	4 (6.6)
40 001-50 000	3 (4.9)
50 001-60 000	4 (6.6)
60 001-70 000	8 (13.1)
70 001-80 000	1 (1.6)
80 001-90 000	4 (6.6)
90 001-100 000	5 (8.2)
100 001-110 000	1 (1.6)
120 001-130 000	4 (6.6)
140 001-150 000	4 (6.6)
150 001-175 000	2 (3.3)
175 001-200 000	1 (1.6)
≥200 001	5 (8.2)
Employment status	
Employed	40 (65.6)
Unemployed	21 (34.4)
Employment type^a^	
Employed, working 40 or more h/wk	27 (44.3)
Employed, working 1-39 h/wk	7 (11.5)
Temporarily employed	1 (1.6)
Self-employed	8 (13.1)
Not employed, looking for work	6 (9.8)
Not employed, not looking for work	1 (1.6)
Homemaker	3 (4.9)
Student (full time)	3 (4.9)
Student (part time)	3 (4.9)
Disabled, not able to work	10 (16.4)
Retired	4 (6.6)
Education level	
High school graduate or equivalent (eg, GED)	4 (6.6)
Trade, technical, or vocational training	2 (3.3)
Some college	8 (13.1)
2-y College degree	4 (6.6)
4-y College degree	15 (24.6)
Master’s degree	22 (36.1)
Doctoral degree	2 (3.3)
Professional degree (JD, MD, MBA)	4 (6.6)

Abbreviation: GED, General Educational Development.

^a^ Participants could select multiple responses, which is why responses do not add up to 61 or 100%.

^b^ Participants were given the option to self-describe their racial and ethnic identity in an open-ended question. Responses included “mixed race” and “Ashkenazi Jewish.”

**Figure.**
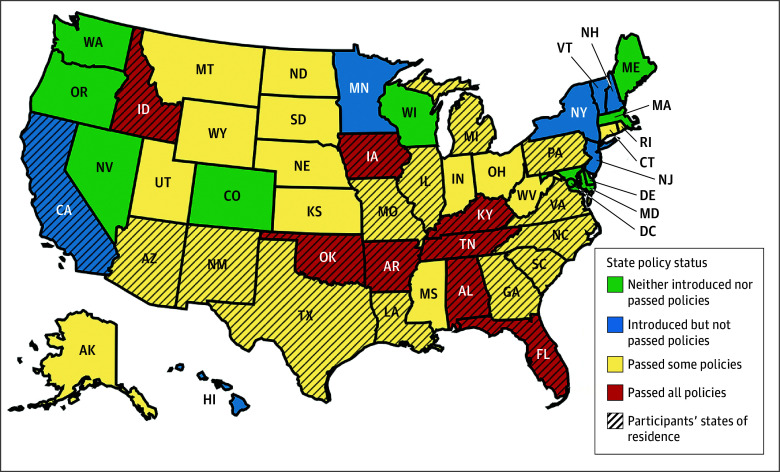
State Sampling Categories and the Geographical Distribution of Participants This figure was developed using the online software program MapChart.net, which is licensed under a Creative Commons Attribution-ShareAlike 4.0 International License.

We organized the perceived mental health impacts of the policies into 3 themes: (1) chronic worry and hypervigilance, (2) social isolation, and (3) hopelessness and powerlessness. We identified a fourth cross-cutting theme: these perceived mental health impacts were felt unequally and more intensely for the most frequent targets of these policies (eg, youths, TNB people), those facing economic insecurity and oppression (eg, racial oppression), and those in less supportive communities (eg, rural and small towns). The 3 themes are discussed in the following sections, with the cross-cutting theme (unequal impacts) discussed within each theme (Table 2 and eTable in Supplement 1).

**Table 2.** Themes and Illustrative Quotes of the Perceived Mental Health Impacts of These Policies

Theme	Illustrative quote (participant and state information)^a^
Chronic worry and hypervigilance	“I think about these policies every day. I think about these policies when my wife has to pee at Walmart. I think about these policies any time we get a job interview, and then no follow up after the actual interview. I think about these policies every time I can’t afford to feed my children because my wife got blacklisted by the [county] public school system for existing while trans on a substitute teaching job where the wrong person was there. It’s a daily fight.” (Participant ID 58, living in a state that passed all policies under study)
	“I have a lot of anxiety about the future…I feel like the walls are narrowing in.” (Participant ID 9, living in a state that passed some but not all policies under study)
Unequal impacts	“My kids are both Black…there’s this extra Southern layer of racism that comes with all of this…particularly for my trans girl. I am not sure I ever fully feel safe when we’re out and about.” (Participant ID 76, living in a state that passed all policies under study)
Social isolation	“They don’t understand why I don’t want to have a close personal relationship with either of them.… I can’t reconcile them saying that they love me and also voting for policies that would make sure that people like me can’t exist…that’s been kind of painful.” (Participant ID 25, living in a state that passed all policies under study)
Unequal impacts	“I want my friends to move back because I can’t afford to move to them.” (Participant ID 5, living in a state that passed all policies under study)
Hopelessness and powerlessness	“I feel like the future is quite bleak.… It’s kind of like, ‘what’s the point?’…the world—it’s kind of falling apart like, ‘What am I fighting for? What am I doing here? Why am I doing my job?’” (Participant ID 44, living in a state that passed some but not all policies under study)
	“Feeling, is it worth it? You’ve done all you can do. You mustered the whole army and the cavalry you thought that did come in with you. You didn’t have enough horses. It feels defeated. And I feel that way sometimes.…I’m feeling battle fatigue.” (Participant ID 8, living in a state that passed some but not all policies under study)
Unequal impacts	“I know more than one trans friend who’s basically just given up on even trying to achieve gender affirming care because…I’ve seen people just give up on trying to achieve the transition that they really want.” (Participant ID 57, living in a state that passed all policies under study)

Abbreviation: ID, identification.

^a^ Quotes throughout the text and in the table have been lightly edited for readability (eg, words such as “like,” “um,” and “yeah” have been removed).

### Chronic Worry and Hypervigilance

Many participants expressed feeling chronically worried and hypervigilant (persistently alert to potential threats)^36,37^ due to the rise of LGBTQIA+-targeted policies. Participants described constantly monitoring their safety: “I’m a lot more hyper aware of my surroundings. My anxiety has gotten worse…from just these bills being proposed.” Several reported worrying about being the targets of harassment daily: “It’s always there in the back of your mind…‘Will this be the day when I have an issue?’ It’s a level of stress on top of the day-to-day stress.” Participants perceived that LGBTQIA+ rights opponents have been licensed to discriminate and are “emboldened by acceptable bigotry to do harm.” Participants described increased anti-LGBTQIA+ violence in their communities as particularly anxiety-inducing.

Although all participants described feeling worried and hypervigilant, they emphasized that these policies disproportionately impact TNB people and youths. Several TNB participants recounted feeling hypervigilant in public spaces: “If I’m in public and I have to go to the bathroom, there’s always that little bit of fear in the back of your head.” Parents expressed persistently fearing their TNB children being rejected or physically unsafe in schools and public spaces. One parent and partner of a TNB person shared:

I think about these policies every day. I think about these policies when my wife has to pee at Walmart. I think about these policies anytime we get a job interview, and then no follow-up after the actual interview. I think about these policies every time I can’t afford to feed my children because my wife got blacklisted by the [county] public school system for existing while trans on a substitute teaching job where the wrong person was there.

Participants shared that LGBTQIA+-targeted policies disproportionately impact people in nonurban areas. Some expressed worrying about their physical safety when leaving cities: “If my car breaks down out here a couple hours outside of Atlanta, am I going to be okay?” Several described researching nonurban communities in advance to determine their safety and concealing their identities in these areas to protect themselves.

Participants explained these policies intensified their fears of economic insecurity and oppression. One participant described facing race- and gender-based employment discrimination before these policies and feeling more vulnerable to discrimination now. Several participants shared incidents of friends losing jobs, particularly in public education, for being outwardly LGBTQIA+ and expressed concerns that they themselves may soon lose employment. Another participant described how policy-related anxiety is compounded by widespread racial discrimination in their communities: “My kids are both Black.… there’s this extra Southern layer of racism that comes with all of this…particularly for my trans girl. I am not sure I ever fully feel safe when we’re out and about.”

All participants, regardless of their identity or context, expressed worry about future policies further restricting their rights. One participant encapsulated this widely felt sentiment, “I have a lot of anxiety about the future.… I feel like the walls are narrowing in.”

### Social Isolation

Participants explained that these policies create social isolation and loneliness. Participants, particularly TNB participants, expressed feeling excised from public life and their communities. One participant shared that a TNB child they know feels cut-off from close friends after being banned from sports teams. Some shared that the need to conceal their identities to protect themselves has been a socially isolating and alienating experience: “I always feel a little otherness…a little wall between me and them.… They can just exist, whereas I have to orchestrate and choreograph.” Several participants, particularly TNB participants, described profound loneliness because they can’t be their authentic selves around others.

These policies have strained participants’ relationships with loved ones and friends. Several mentioned severing ties with family to protect their well-being. One TNB participant described reckoning with parental rejection:

They don’t understand why I don’t want to have a close personal relationship with either of them.… I can’t reconcile them saying that they love me and also voting for policies that would make sure that people like me can’t exist…that’s been kind of painful.

These policies have licensed people in participants’ lives to share their anti-LGBTQIA+ attitudes, which has prompted some participants to withdraw from even close friends: “Specifically for trans youth healthcare…I try to avoid talking about any personal matters with friends.”

Participants described loved ones fleeing states with LGBTIQA+-targeted policies, which has fractured communities and isolated those that remain. Several expressed dismay about losing their social support networks: “I’ve had friends leave the state. That has been hard, because I miss them.” Several explicitly mentioned GAC bans as driving people to leave states. TNB participants expressed frustration that they must choose between accessing life-saving health care and remaining in their communities:

It’s been really, really difficult…trying to weigh my love and my attachment for my home and my ability to live safely and happily, and with the medical treatment that I need to survive…this is my home, it’s so beautiful, and I deserve to be here.

Participants shared that the social isolation resulting from these policies affects economically disadvantaged people more. Several described financial barriers to remaining near friends because states with protective policies tend to have higher living costs:^38^ “I want my friends to move back because I can’t afford to move to them.” Others expressed feeling left behind to combat these policies: “It is the people of privilege that are leaving. And that’s very frustrating to me…who’s gonna help continue to fight?”

### Hopelessness and Powerlessness

Many participants expressed feeling powerless about the future due to the rise of LGBTQIA+-targeted policies. Several described profound despair: “I feel like the future is quite bleak.… It’s kind of like, ‘what’s the point?’…the world—it’s kind of falling apart like, ‘What am I fighting for? What am I doing here? Why am I doing my job?’” The sheer number of policies makes many feel emotionally exhausted, “defeated,” and “battle fatigue[d]” about combating them. Even participants heavily engaged in LGBTQIA+ advocacy expressed losing hope in organizing: “Feeling, is it worth it? You’ve done all you can do. You mustered the whole army and the cavalry.… You didn’t have enough horses.” Several discussed feeling depleted from their advocacy, like one parent opposed to their state’s public education restrictions: “My kids, they’ve basically been given…propaganda that has been approved by the state as legitimate education material. Fighting that…has been difficult.”

Many participants, especially TNB participants and rural participants, described perceiving their desired futures as foreclosed—that the lives they want no longer feel possible. Growing GAC inaccessibility has left many TNB participants feeling hopeless about ever medically transitioning: “I know more than one trans friend who’s basically just given up on even trying to achieve gender affirming care.” Another participant reflected that transitioning also feels futile because of the political situation:

Now I feel like it doesn’t matter how well you pass.… It doesn’t matter how much effort you put into it…enough people are so mad and so heated that it doesn’t matter. “You are never going to be what you want to be, and we are going to hate you.”

Some are reconsidering their long-term life plans, like their pursuits of specific careers and desires to live in non-urban areas: “Moving out of the 20-minute radius of St. Louis, I feel a lot less safe. I am thinking about studying library sciences…thinking about how librarians and teachers could be penalized for talking about queer people, that is really threatening and scary.” Several participants are pessimistic about the future due to these policies:

There was a time that I was involved in politics and felt very hopeful…realizing I was trans and beginning transition made me feel very hopeful and excited about the future. A lot of that excitement was lost, especially when policies became a reality that were restricting anybody’s access to trans health care…now it’s just a slippery slope down into having to enact our emergency plan, and nobody likes to make that their thing they’re thinking toward in the future. People want to grow and flourish and find new projects and grow their communities and not think about, “What do I do if there’s a proverbial earthquake?”

Across interviews, participants consistently shared that the outcome of the 2024 presidential election would determine whether to hold out hope for the future.

## Discussion

Our qualitative analysis highlights the profound psychological distress experienced by diverse LGBTQIA+ people living in states where LGBTQIA+-targeted policies have been proposed or enacted. We identified 3 mental health themes and 1 cross-cutting theme regarding these policies’ perceived impacts. First, participants described feeling chronically worried and hypervigilant due to fears of increased discrimination, harassment, violence, loss of employment and health care, and further abrogation of their rights. Second, participants expressed that these policies impose barriers to participation in public life, weaken community support by prompting some to move to safer places, and complicate existing relationships with loved ones and friends—resulting in social isolation. Third, participants underscored that these policies’ rapid proliferation has left them feeling hopeless and powerless about the future. Finally, participants emphasized that these policies’ negative psychological impacts seem intensified for those most targeted (youths, TNB people) and those with limited social and economic resources (economically disadvantaged people, racially and ethnically minoritized people, and those in rural areas without LGBTQIA+ social support networks).

Our results support existing quantitative work finding associations between LGBTQIA+-targeted policies and worse mental health^6,8,39,40,41^ and provide a rich description of the complex and heterogenous psychosocial processes through which LGBTQIA+ people experience these policies. By centering LGBTQIA+ people’s perspectives, we provide an inductive exploration of these processes as they unfold in people’s lives. LGBTQIA+ people perceive LGBTQIA+-targeted policies as directly impacting them by threatening their safety and depriving them of basic rights and access to public life and as indirectly impacting them by emboldening bigotry, fracturing communities, and lowering people’s future expectations. Participants’ experiences reinforce emerging research informed by minority stress and social safety theories on the psychosocial processes mediating the association between LGBTQIA+-targeted policies and LGBTQIA+ people’s mental health, including the role of hypervigilance,^37^ social isolation and social safety loss,^11,14^ identity concealment,^42^ and hopelessness due to foreclosed futures.^43^ Our findings that LGBTQIA+ people do not experience these policies uniformly—policies compound and amplify other structural inequities (economic inequality, racial oppression)—are consistent with the integrative perspective of the social determinants of health framework.^44,45^

Our findings provide future research directions. First, as more states and the federal government target LGBTQIA+ rights, investigating the first-hand accounts and mental health experiences of LGBTQIA+ people in states with and without LGBTQIA+-targeted state policies is crucial. LGBTQIA+ people in states without these policies may experience spillover effects from other states’ policies while navigating an increasingly discriminatory federal policy environment.^46^ Second, our findings suggest that LGBTQIA+-targeted policies are felt unequally by LGBTQIA+ people and that there is an iterative relationship between these policies and other structural inequities. Preexisting inequities compound and amplify LGBTQIA+-targeted policies’ health-related implications (eg, unemployment exacerbates GAC bans for TNB adults who cannot afford GAC), and LGBTQIA+-targeted policies have cascading structural implications (eg, these policies license employment discrimination against TNB people). This dialectical relationship between structural inequities is rarely captured in quantitative studies that assume structural factors are static and separable. Qualitative approaches remain best-suited for the task of capturing these dynamic, indivisible, and iterative processes.^47,48,49,50^ Future qualitative research should continue to examine how these structural processes influence health from the perspectives of those who experience them directly.

The policy environment for LGBTQIA+ people is changing rapidly.^5,51,52^ Beyond LGBTQIA+-targeted policies, many recent federal policies will likely harm the health of most US residents (eg, Medicaid cuts, federal agency and program cuts) and disproportionately affect the socially and economically vulnerable—compounding the negative consequences of LGBTQIA+-specific policies. These policies will prevent researchers, clinicians, and advocates from developing and implementing multilevel interventions to support population mental health. There is an urgent need to sustain public health infrastructure and health services for all while simultaneously providing rapid and targeted multilevel support for LGBTQIA+ people amid this dynamic policy environment.

### Limitations

Our study has limitations. Although parents shared their perspectives on how these policies have influenced their children’s lives, we did not directly seek the perspectives of youth—the target of many policies. Our interviews also took place during the months preceding the 2024 US presidential election—a period characterized by political volatility and uncertainty. This uncertainty may have amplified participants’ anticipatory anxiety, compounding the psychological distress they were experiencing from state LGBTQIA+-targeted policies. Thus, our findings capture the psychological experiences of LGBTQIA+ adults living in states with LGBTQIA+-targeted policies in the context of a broader, shifting national political landscape.

## Conclusions

In this qualitative study of 61 LGBTQIA+ adults, participants expressed feelings of chronic worry and hypervigilance, social isolation, and hopelessness and powerlessness following the proposal and/or enactment of state policies targeting LGBTQIA+ rights. Participants perceived the policies to more profoundly impact those most targeted (youths, TNB people) and to amplify preexisting economic, racial, and geographic inequities. Qualitative research is essential to understand LGBTQIA+ people’s perspectives at a time when state and federal policies targeting LGBTQIA+ rights have increased.

## Notes

Supplement 1. **eTable.** Themes and Full List of Participant Quotes of Policies’ Perceived Mental Health Impacts

Supplement 2.
Data Sharing Statement
