# Supplementary material for: LGBTQIA+ People’s Perspectives on LGBTQIA+-Targeted State Policies and Mental Health: A Qualitative Study
Source: JAMA Netw Open. 2026 Jan 2;9(1):e2546538. doi: 10.1001/jamanetworkopen.2025.46538 (PMC12761330; doi:10.1001/jamanetworkopen.2025.46538)
Supplement: Supplement 1. — eTable. Themes and Full List of Participant Quotes of Policies’ Perceived Mental Health Impacts [file jamanetwopen-e2546538-s001.pdf]

Supplemental Online Content

Last BS, Poupard M, Williamson N, et al. LGBTQIA+ people's perspectives on LGBTQIA+-targeted state policies and mental health: a qualitative study. *JAMA Netw Open*. 2025;8(12):e2546538. doi:10.1001/jamanetworkopen.2025.46538

eTable. Themes and Full List of Participant Quotes of Policies' Perceived Mental Health Impacts

This supplemental material has been provided by the authors to give readers additional information about their work.

© 2025 Last BS et al. *JAMA Network Open*.

eTable 1. Themes and Full List of Participant Quotes of Policies' Perceived Mental Health Impacts.

Theme	Illustrative Quote
**Chronic Worry and Hypervigilance**	"I'm a lot more hyper aware of my surroundings. My anxiety has gotten worse…from just these bills being proposed." (Participant ID 20, living in a state that passed all policies under study)<br><br>"It's always there in the back of your mind…'Will this be the day when I have an issue?' It's a level of stress on top of the day-to-day stress." (Participant ID 3, living in a state that passed all policies under study)<br><br>"It's fears of people's actions, and people being emboldened by acceptable bigotry to do harm." (Participant ID 20, living in a state that passed all policies under study)<br><br>"If I'm in public and I have to go to the bathroom, there's always that little bit of fear in the back of your head." (Participant ID 75, living in a state that passed all policies under study).<br><br>"I think about these policies every day. I think about these policies when my wife has to pee at Walmart. I think about these policies any time we get a job interview, and then no follow-up after the actual interview. And I think about these policies every time I can't afford to feed my children because my wife got blacklisted by the [county] public school system for existing while trans on a substitute teaching job where the wrong person was there." (Participant ID 58, living in a state that passed all policies under study)<br><br>"If my car breaks down out here a couple hours outside of Atlanta, am I going to be okay?" (Participant ID 9, living in a state that passed some but not all policies under study)<br><br>"I have a lot of anxiety about the future…I feel like the walls are narrowing in." (Participant ID 9, living in a state that passed some but not all policies under study)
Unequal Impacts	"My kids are both Black…there's this extra Southern layer of racism that comes with all of this…particularly for my trans girl. I am not sure I ever fully feel safe when we're out and about." (Participant ID 76, living in a state that passed all policies under study)
**Social Isolation**	"I always feel a little otherness…a little wall between me and them…They can just exist, whereas I have to orchestrate and choreograph." (Participant ID 58, living in a state that passed all policies under study)<br><br>"They don't understand why I don't want to have a close personal relationship with either of them…I can't reconcile them saying that they love me and also voting for policies that would make sure that people like me can't exist…that's been kind of painful." (Participant ID 25, living in a state that passed all policies under study)

© 2025 Last BS et al. *JAMA Network Open*.

	“Specifically for trans youth healthcare…I try to avoid talking about any personal matters with friends.” (Participant ID 24, living in a state that passed some but not all policies under study)<br><br>“I've had friends leave the state. And that has been hard, because I miss them.” (Participant ID 31, living in a state that passed all policies under study)<br><br>“It's been really, really difficult…trying to weigh my love and my attachment for my home and my ability to live safely and happily, and with the medical treatment that I need to survive…this is my home, and it's so beautiful and I deserve to be here.” (Participant ID 21, living in a state that passed all policies under study)
Unequal Impacts	“I want my friends to move back because I can't afford to move to them.” (Participant ID 5, living in a state that passed all policies under study)<br><br>“It is the people of privilege that are leaving. And that's very frustrating to me…who's gonna help continue to fight?” (Participant ID 77, living in a state that passed all policies under study)
**Hopelessness and Powerlessness**	“I feel like the future is quite bleak…It's kind of like, ‘what's the point?’…the world—it's kind of falling apart like, ‘What am I fighting for? What am I doing here? Why am I doing my job?’” (Participant ID 44, living in a state that passed some but not all policies under study)<br><br>“Feeling, is it worth it? You've done all you can do. You mustered the whole army and the cavalry you thought that did come in with you. You didn't have enough horses. It feels defeated. And I feel that way sometimes…I'm feeling battle fatigue.” (Participant ID 8, living in a state that passed some but not all policies under study)<br><br>“My kids, they've basically been given…propaganda that has been approved by the state as legitimate education material. Fighting that…has been difficult.” (Participant ID 3, living in a state that passed all policies under study)<br><br>“Now I feel like it doesn't matter how well you pass…It doesn't matter how much effort you put into it…enough people are so mad and so heated that it doesn't matter. ‘You are never going to be what you want to be, and we are going to hate you.’” (Participant ID 46, living in a state that passed some but not all policies under study)<br><br>“Moving out of the 20-minute radius of St. Louis, I feel a lot less safe. I am thinking about studying library sciences…thinking about how librarians and teachers could be penalized for talking about queer people, that is really threatening and scary.” (Participant ID 22, living in a state that passed some but not all policies under study)<br><br>“There was a time that I was involved in politics and felt very hopeful about all of that…realizing I was trans and beginning transition made me feel very hopeful and excited about the future. And a lot of that excitement was lost, especially when policies became a reality that were restricting anybody's access to trans health care…now it's just a slippery slope down into having to enact our emergency plan, and nobody likes to make that their thing they're thinking toward in the future.

© 2025 Last BS et al. *JAMA Network Open*.

	People want to grow and flourish and find new projects and grow their communities and not think about, 'What do I do if there's a proverbial earthquake?'" (Participant ID 51, living in a state that passed some but not all policies under study)
Unequal Impacts	"I know more than one trans friend who's basically just given up on even trying to achieve gender affirming care" (Participant ID 57, living in a state that passed all policies under study)

Note. Quotes throughout the text and in the table have been lightly edited for readability (*e.g.*, words such as "like," "um," and "yeah" have been removed).

© 2025 Last BS et al. *JAMA Network Open*.
