# Supplementary material for: LGBTQIA+ People’s Perspectives on LGBTQIA+-Targeted State Policies and Mental Health: A Qualitative Study
Source: JAMA Netw Open. 2026 Jan 2;9(1):e2546538. doi: 10.1001/jamanetworkopen.2025.46538 (PMC12761330; doi:10.1001/jamanetworkopen.2025.46538)
Supplement: Supplement 2. — Data Sharing Statement [file jamanetwopen-e2546538-s002.pdf]

# Data Sharing Statement

Last. LGBTQIA+ People’s Perspectives on LGBTQIA+-Targeted State Policies and Mental Health. *JAMA Netw Open*. Published December 03, 2025.
doi:10.1001/jamanetworkopen.2025.46538

**Data**
**Data available:** No

**Additional Information**
**Explanation for why data not available:** Due to the sensitivity of qualitative data and ethical restrictions related to sensitive participant information, study data will not be made available.
